# Supplementary figures and images for: A hierarchical pathway for assembly of the distal appendages that organize primary cilia
Source: eLife. 2025 Jan 30;14:e85999. doi: 10.7554/eLife.85999 (PMC11984956; doi:10.7554/eLife.85999)

Figure 1-figure supplement 2B\_CEP83

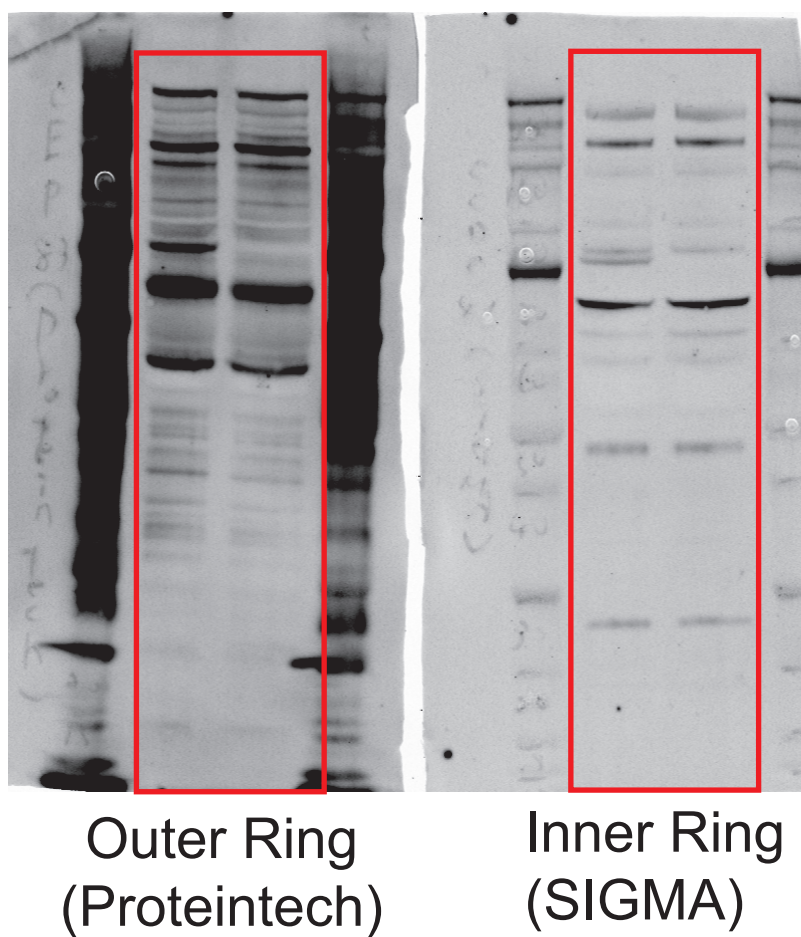

Supplement: Figure 1—figure supplement 2—source data 2. [file elife-85999-fig1-figsupp2-data2.pdf]

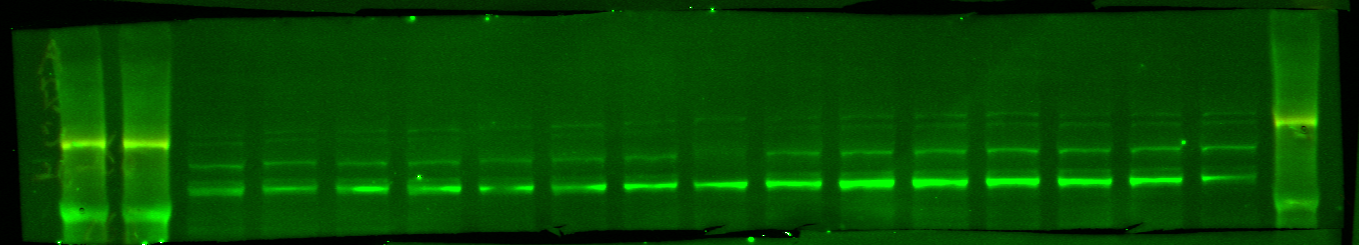

Supplement: Figure 2—figure supplement 2—source data 1. [file elife-85999-fig2-figsupp2-data1.zip › Figure 2-figure supplement 1-Source Data/Figure 2-figure supplement 1-Source Data 1/ANKRD26.tif]

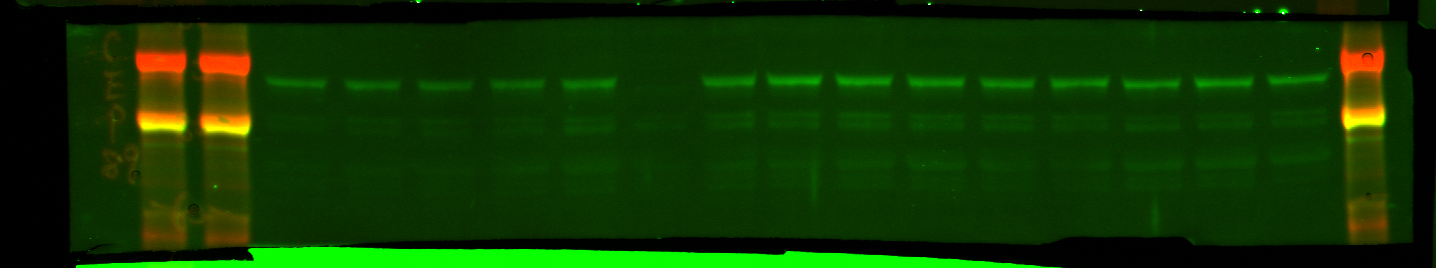

Supplement: Figure 2—figure supplement 2—source data 1. [file elife-85999-fig2-figsupp2-data1.zip › Figure 2-figure supplement 1-Source Data/Figure 2-figure supplement 1-Source Data 1/CEP89.tif]

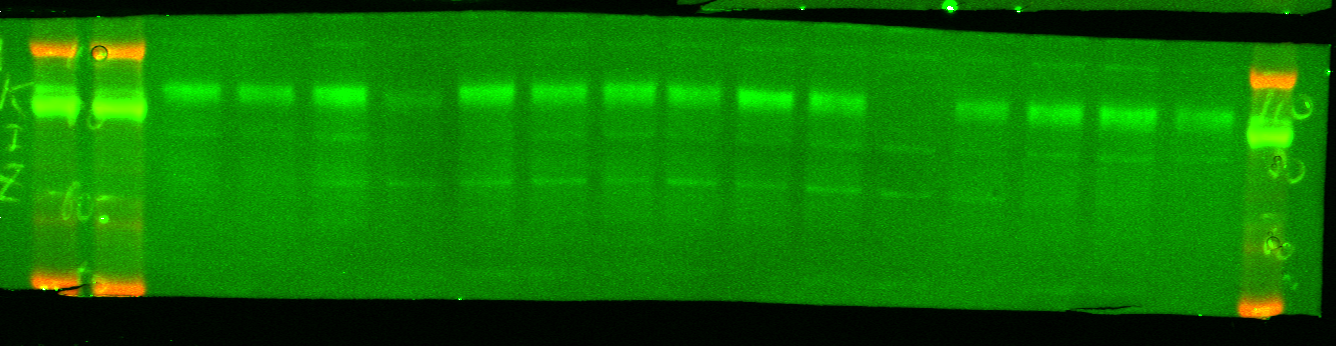

Supplement: Figure 2—figure supplement 2—source data 1. [file elife-85999-fig2-figsupp2-data1.zip › Figure 2-figure supplement 1-Source Data/Figure 2-figure supplement 1-Source Data 1/KIZ.tif]

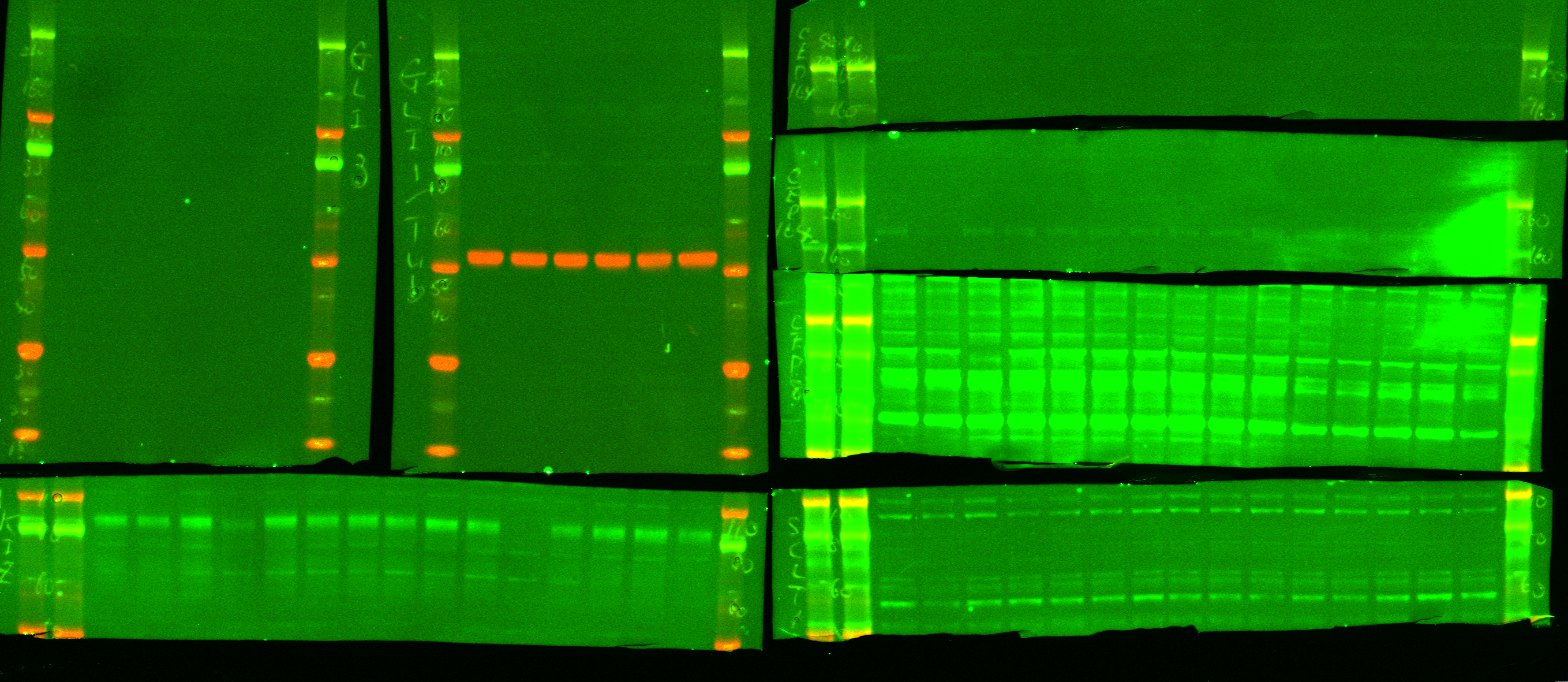

Supplement: Figure 2—figure supplement 2—source data 1. [file elife-85999-fig2-figsupp2-data1.zip › Figure 2-figure supplement 1-Source Data/Figure 2-figure supplement 1-Source Data 1/CEP164.tif]

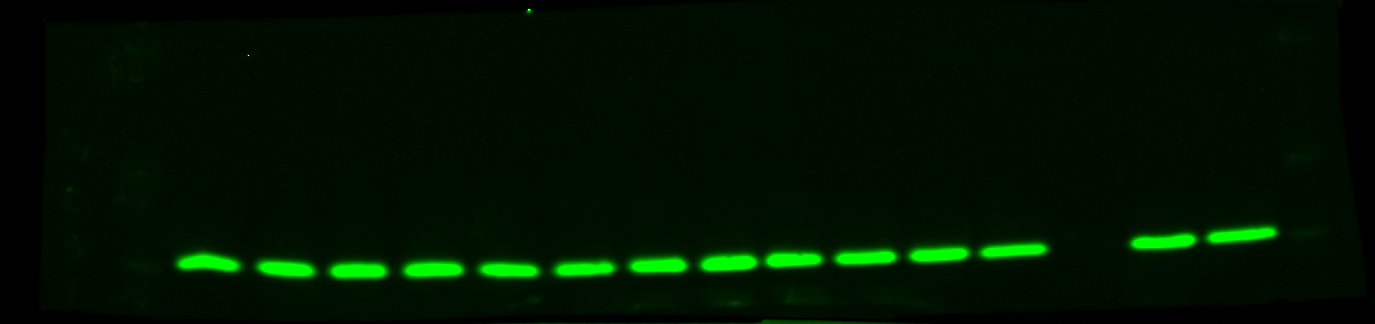

Supplement: Figure 2—figure supplement 2—source data 1. [file elife-85999-fig2-figsupp2-data1.zip › Figure 2-figure supplement 1-Source Data/Figure 2-figure supplement 1-Source Data 1/NCS1.tif]

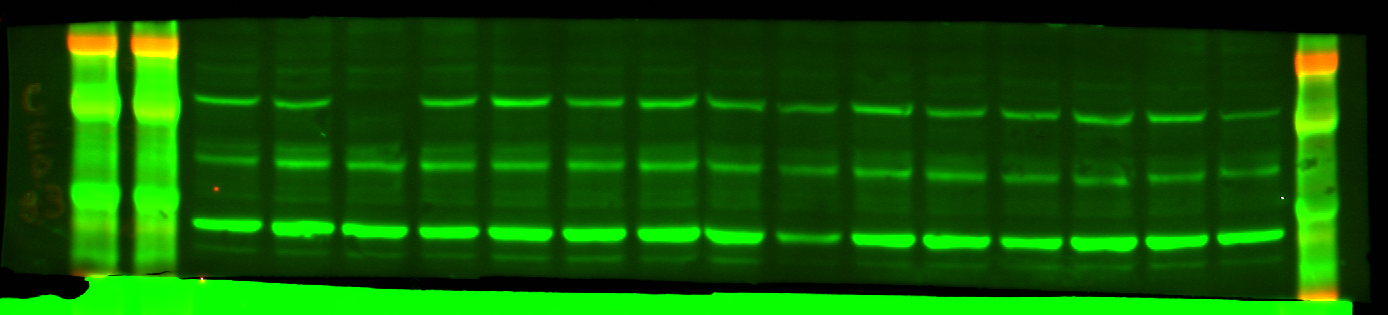

Supplement: Figure 2—figure supplement 2—source data 1. [file elife-85999-fig2-figsupp2-data1.zip › Figure 2-figure supplement 1-Source Data/Figure 2-figure supplement 1-Source Data 1/CEP83.tif]

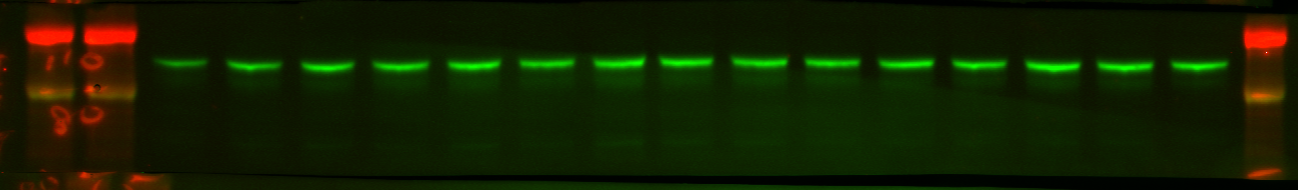

Supplement: Figure 2—figure supplement 2—source data 1. [file elife-85999-fig2-figsupp2-data1.zip › Figure 2-figure supplement 1-Source Data/Figure 2-figure supplement 1-Source Data 1/IFT88.tif]

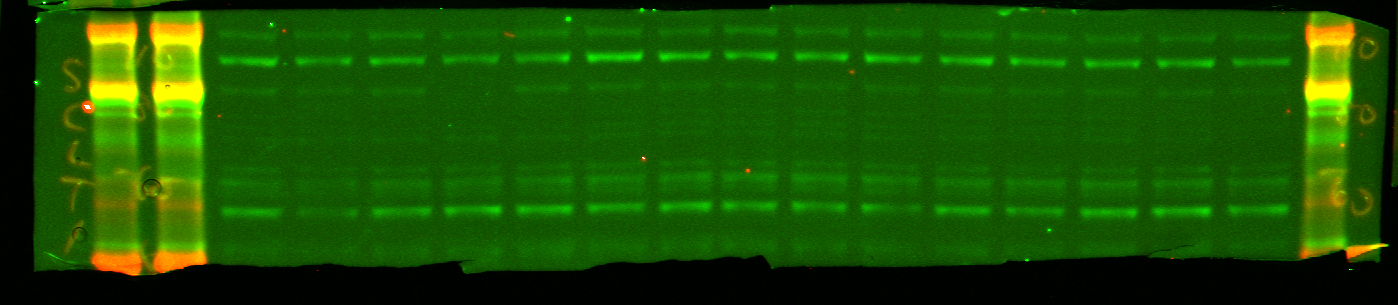

Supplement: Figure 2—figure supplement 2—source data 1. [file elife-85999-fig2-figsupp2-data1.zip › Figure 2-figure supplement 1-Source Data/Figure 2-figure supplement 1-Source Data 1/SCLT1.tif]

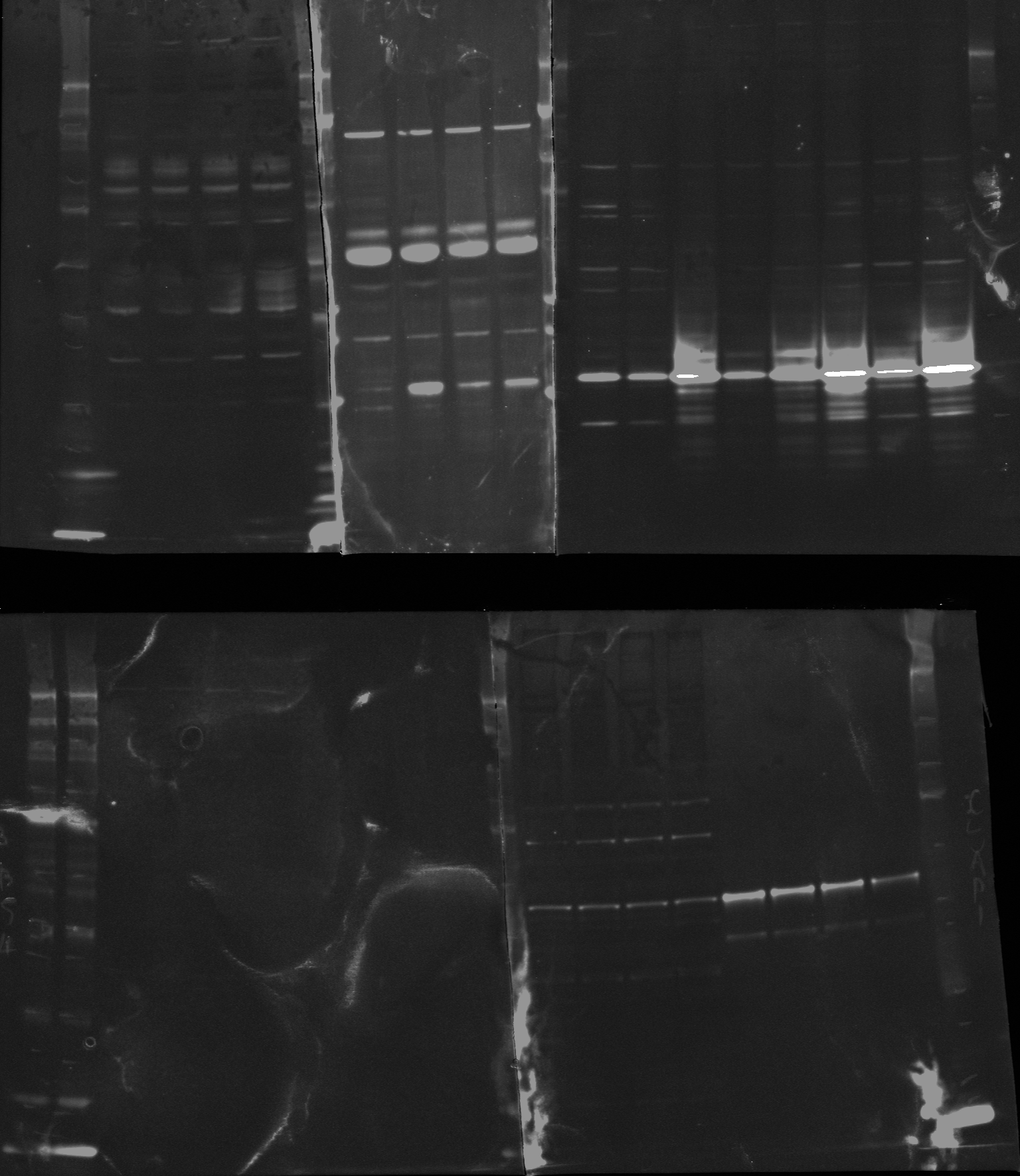

Supplement: Figure 4—figure supplement 2—source data 1. [file elife-85999-fig4-figsupp2-data1.zip › Figure 4-figure supplement 2-Source Data 1/IFT52_FLAG_Rabl2b_BBS4_CLUAP1Grayscale800.TIF]

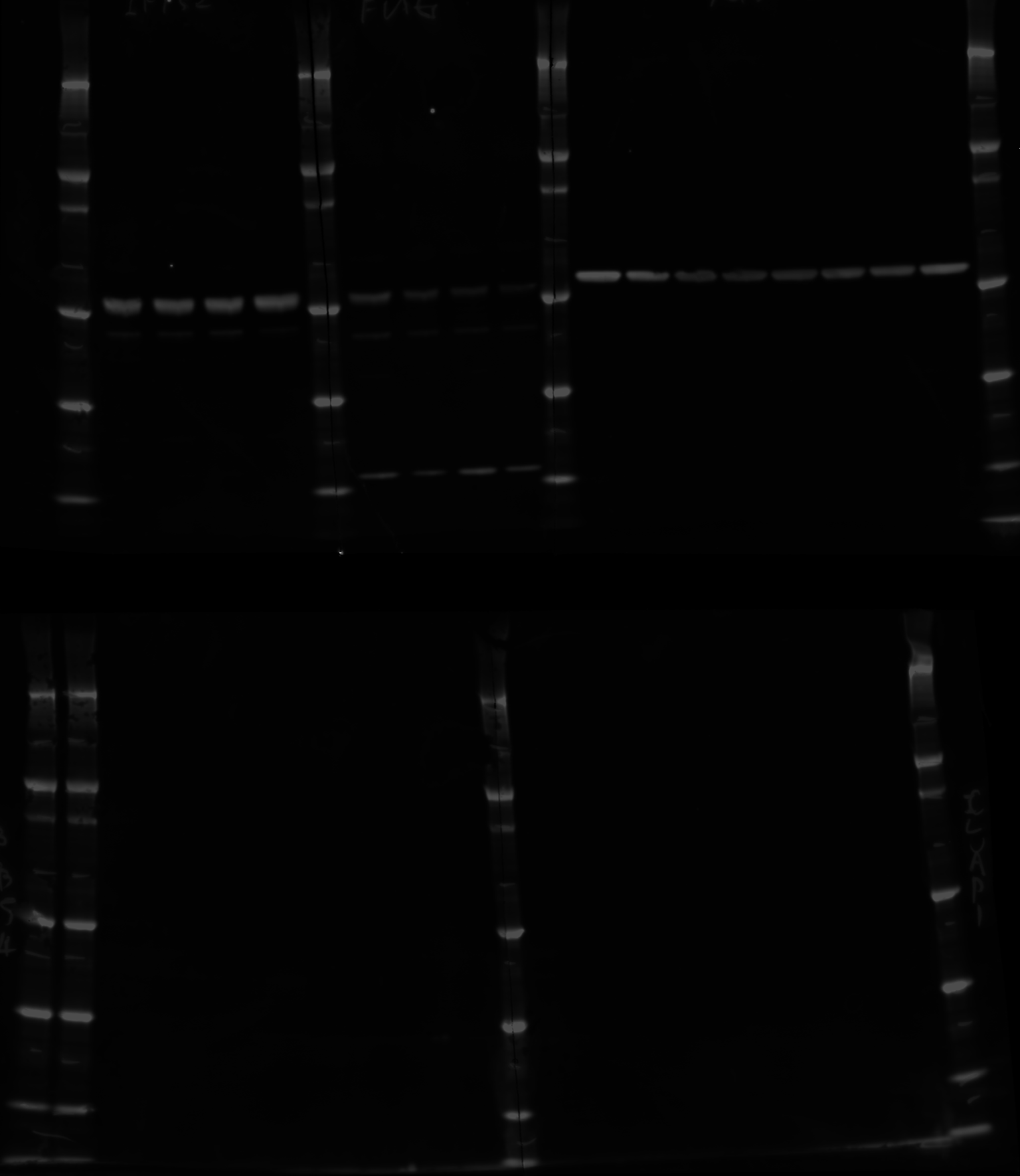

Supplement: Figure 4—figure supplement 2—source data 1. [file elife-85999-fig4-figsupp2-data1.zip › Figure 4-figure supplement 2-Source Data 1/Tubulin_Grayscale700.TIF]
