# Supplementary material for: A hierarchical pathway for assembly of the distal appendages that organize primary cilia
Source: eLife. 2025 Jan 30;14:e85999. doi: 10.7554/eLife.85999 (PMC11984956; doi:10.7554/eLife.85999)
Supplement: Figure 2—figure supplement 2—source data 1. [file elife-85999-fig2-figsupp2-data1.zip › Figure 2-figure supplement 1-Source Data/Figure 2-figure supplement 2-Source Data 2.pdf]

Figure 2-figure supplement 2A_CEP164

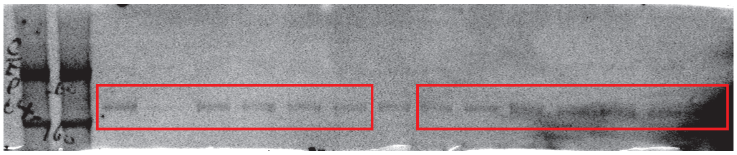

Figure 2-figure supplement 2A_CEP89

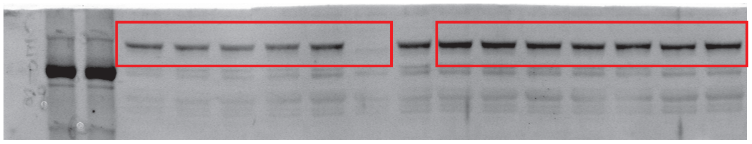

Figure 2-figure supplement 2A_CEP83

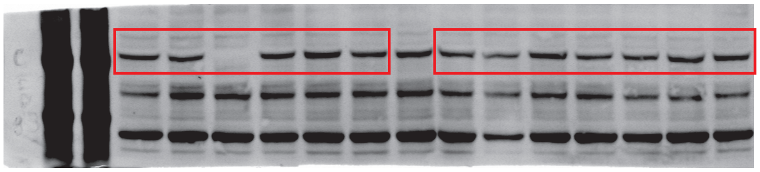

Figure 2-figure supplement 2A_ANKRD26

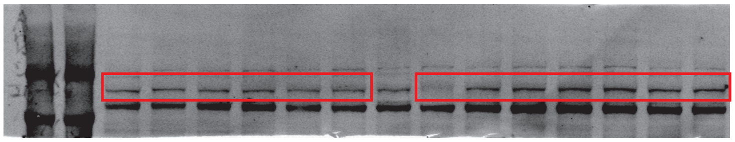

Figure 2-figure supplement 2A_KIZ

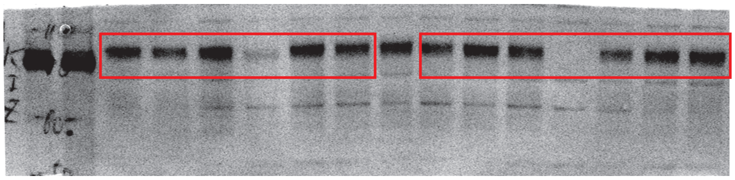

Figure 2-figure supplement 2A_NCS1

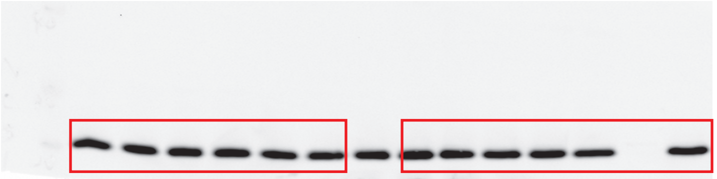

Figure 2-figure supplement 2A_SCLT1

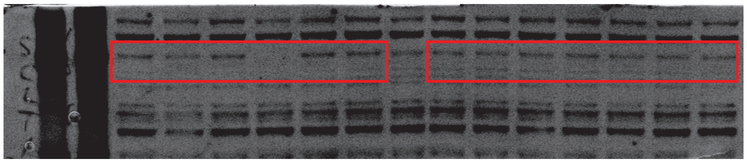

Figure 2-figure supplement 2A_IFT88

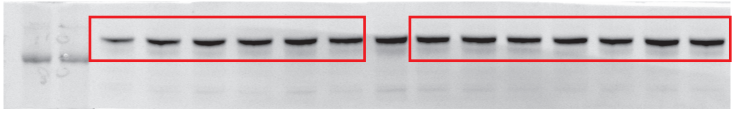

Kanie et al., Figure 2-figure supplement 2-Source Data 2
