# Supplementary material for: A hierarchical pathway for assembly of the distal appendages that organize primary cilia
Source: eLife. 2025 Jan 30;14:e85999. doi: 10.7554/eLife.85999 (PMC11984956; doi:10.7554/eLife.85999)
Supplement: Figure 4—figure supplement 1—source data 2. [file elife-85999-fig4-figsupp1-data2.pdf]

Figure 4-figure supplement 1A_RAB34

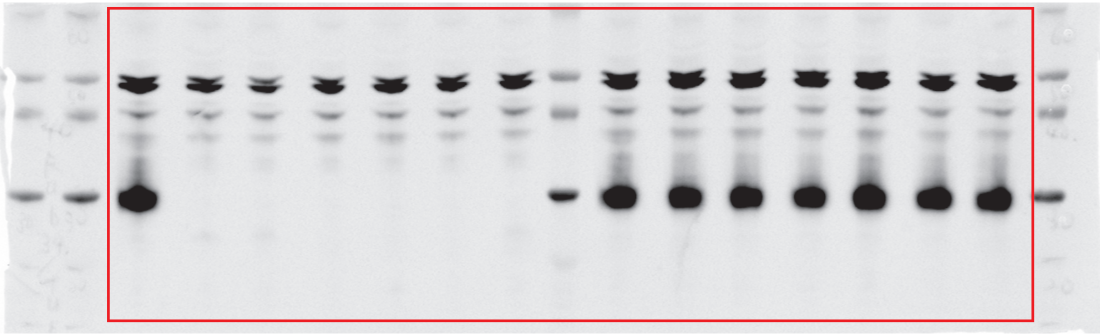

Figure 4-figure supplement 1A_MYO5A

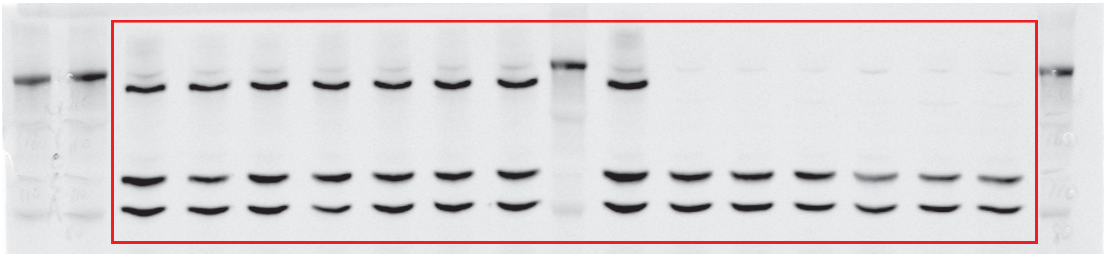

Figure 4-figure supplement 1A_Tubulin

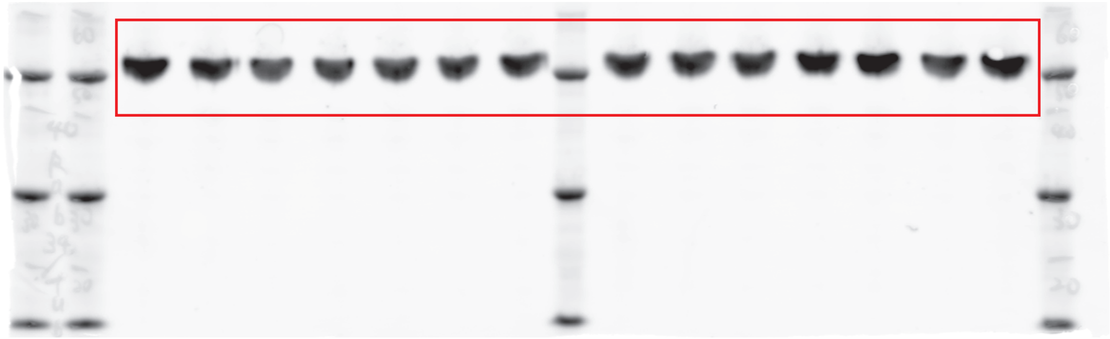

Kanie et al., Figure 4-figure supplement 1-Source Data 2
