# Supplementary material for: A hierarchical pathway for assembly of the distal appendages that organize primary cilia
Source: eLife. 2025 Jan 30;14:e85999. doi: 10.7554/eLife.85999 (PMC11984956; doi:10.7554/eLife.85999)
Supplement: Source data 2. [file elife-85999-data2.pdf]

```
current_file = getTitle() ;

run("Duplicate...", "title=centrosome duplicate channels=3");
roiManager("reset") ;

//cep170 mask
selectWindow("centrosome") ;
run("Subtract Background...", "rolling=5");
setThreshold(70, 65535);
setOption("BlackBackground", true);
run("Convert to Mask");

//cep83 mask
selectWindow(current_file) ;
run("Duplicate...", "title=cep19 duplicate channels=2");
selectWindow("cep19") ;
run("Subtract Background...", "rolling=5");
setForegroundColor(255,255,255);
setThreshold(180,65535);
setOption("BlackBackground",true) ;
run("Convert to Mask") ;
run("Open") ;


//combination mask
run("Images to Stack","name=combo_mask");
run("Z Project...","projection=[Max Intensity]") ;
run("Dilate") ;
run("Dilate");
run("Dilate");
run("Dilate");
run("Dilate");

run("Erode") ;
run("Erode") ;
run("Erode") ;

selectWindow(current_file)  ;
Stack.setChannel(2) ;
run("Duplicate...","title=CEP83_"+current_file+" channels=2") ;
run("Subtract Background...","rolling=5") ;

selectWindow("MAX_combo_mask") ;
run("ROI Manager...");
```

```
run("Set Measurements...", "area mean min centroid shape integrated display
redirect=CEP83_"+current_file+" decimal=3");
run("Analyze Particles...", "size=50-Infinity pixel circularity=0.55-1.00 exclude add");
roiManager("Measure");
selectWindow("CEP83_"+current_file) ;
close() ;
roiManager("Delete");


//clean up
selectWindow("MAX_combo_mask") ;
close() ;
selectWindow("combo_mask");
close() ;
```
