# Supplementary material for: A hierarchical pathway for assembly of the distal appendages that organize primary cilia
Source: eLife. 2025 Jan 30;14:e85999. doi: 10.7554/eLife.85999 (PMC11984956; doi:10.7554/eLife.85999)
Supplement: Source data 3. [file elife-85999-data3.pdf]

```
current_file = getTitle() ;

run("Duplicate...", "title=cilia duplicate channels=2");
roiManager("reset") ;


//ARL13B mask
selectWindow("cilia") ;
run("Subtract Background...", "rolling=5");
setThreshold(140, 65535);
setOption("BlackBackground", true);
run("Convert to Mask");


run("Dilate");
run("Dilate");
run("Dilate");
run("Erode") ;
run("Erode") ;

run("Erode") ;


selectWindow(current_file)  ;
Stack.setChannel(2) ;
run("Duplicate...","title=ARL13B_"+current_file+" channels=2") ;
run("Subtract Background...","rolling=5") ;

selectWindow("cilia") ;
run("ROI Manager...");
run("Set Measurements...", "area mean min centroid shape integrated display
redirect=ARL13B_"+current_file+" decimal=3");
run("Analyze Particles...", "size=10-infinity pixel circularity=0-0.5 exclude add");
roiManager("Measure");
selectWindow("ARL13B_"+current_file) ;
close() ;
roiManager("Delete");

//clean up
selectWindow("cilia") ;
close() ;
```
